# The 6R’s of drug induced nephrotoxicity

**DOI:** 10.1186/s12882-017-0536-3

**Published:** 2017-04-03

**Authors:** Linda Awdishu, Ravindra L. Mehta

**Affiliations:** 1 UC San Diego Skaggs School of Pharmacy, San Diego, USA; 2 UC San Diego School of Medicine, 9500 Gilman Dr, La Jolla, CA 92093 USA

**Keywords:** Nephrotoxicity, Acute kidney injury, Drugs, Hypersensitivity, Adverse reaction, Tubular toxicity, Nephrolithiasis, Glomerular, Crystalluria

## Abstract

Drug induced kidney injury is a frequent adverse event which contributes to morbidity and increased healthcare utilization. Our current knowledge of drug induced kidney disease is limited due to varying definitions of kidney injury, incomplete assessment of concurrent risk factors and lack of long term outcome reporting. Electronic surveillance presents a powerful tool to identify susceptible populations, improve recognition of events and provide decision support on preventative strategies or early intervention in the case of injury. Research in the area of biomarkers for detecting kidney injury and genetic predisposition for this adverse event will enhance detection of injury, identify those susceptible to injury and likely mitigate risk. In this review we will present a 6R framework to identify and mange drug induced kidney injury – risk, recognition, response, renal support, rehabilitation and research.

## Background

Drug-induced nephrotoxicity is increasingly recognized as a significant contributor to kidney disease including acute kidney injury (AKI) and chronic kidney disease (CKD). Nephrotoxicity has a wide spectrum, reflecting damage to different nephron segments based upon individual drug mechanisms. Both glomerular and tubular injuries are recognized targets for drug toxicity and may result in acute or chronic functional changes. However, standard definitions of drug induced kidney disease (DIKD) are lacking, leading to challenges in recognition and reporting. The clinical manifestations of DIKD often go unrecognized, particularly in the setting of short drug exposures. This poses challenges in assessing the incidence, severity and long-term consequences of DIKD.

Our knowledge of the epidemiology of nephrotoxicity focuses predominantly on drug induced AKI. Prospective cohort studies of AKI have documented the frequency of drug-induced nephrotoxicity to be approximately 14-26% in adult populations [1–3]. Nephrotoxicity is a significant concern in pediatrics with 16% of hospitalized AKI events being attributable primarily to a drug [4]. The epidemiology of tubular disorders is unclear as a standard definition is lacking and many published reports document tubular dysfunction leading to AKI. This may under-estimate the true incidence of tubular disorders since only cases associated with a change in serum creatinine (Scr) are recognized. However, frequent use of specific drugs, such as tenofovir, has led to greater attention to tubular injuries with documented frequencies of 12–22% of treated subjects in cohort studies [5, 6]. Glomerular injury is uncommon and most of the literature is limited to case reports or case series. However, novel chemotherapeutic agents are increasingly being associated with this form of toxicity [7]. Given these challenges in the reported epidemiology and outcomes of DIKD, we propose a novel framework to approach drug induced nephrotoxicity focused on Risk assessment, early Recognition, targeted Response, timely Renal support and Rehabilitation coupled with Research (the 6R approach).

## Risk

To evaluate the risk of nephrotoxicity, general questions can be applied to each causal drug. What is the predictable risk based on the known pharmacology of the drug? What is the known risk, contributing risk factors and the typical timeline for injury? If the risks are known, how is this information used clinically to predict the risk for an individual patient (i.e. clinical risk scores for contrast nephropathy)? How is this information used to mitigate the risk?

Drug induced adverse events can be classified into two categories: dose dependent and idiosyncratic reactions. This categorization is important to consider in the context of drug induced kidney disease (DIKD) since the mechanisms for drug toxicity are different posing challenges in risk assessment. Dose dependent reactions are predictable from the known pharmacology of the drug. For example, the risk of aminoglycoside induced nephrotoxicity increases with higher trough drug concentrations and longer duration of therapy [8]. Whereas, interstitial nephritis from proton pump inhibitors is an unpredictable idiosyncratic reaction, which is unlikely to be preventable or minimized.

When assessing the known risk of DIKD, often this information may be in the form of case reports, adverse event reporting from clinical trials or post marketing surveillance [9]. Prospective studies focused on determining the incidence of DIKD are few. Most studies are retrospective and are focused largely on drugs with predictable toxicities and therapeutic drug monitoring (TDM). Determining the incidence of idiosyncratic reactions is difficult since data is often limited to case reports. Some studies attempt to demonstrate an association using claims data and diagnostic codes; however, the incidence of the AKI is variable between cohorts and likely overstated [10–12] Most importantly, the definition of DIKD has not been standardized, making interpretation of the epidemiology challenging. The information on drug specific DIKD risk is summarized in Table 1.

**Table 1:** 5R Summary by Causal Drug

Drug/Phenotype	Risk			Recognition	Response	Renal support	Rehabilitation
	Patient specific	Disease specific	Process of care				
Aminoglycosides [8, 74–76] AKI	Age	Diabetes Volume depletion Sepsis Liver dysfunction CKD Hypokalemia Hypomagnesemia	Duration of therapy Type of aminoglycoside Frequency of dosing Elevated trough concentrations > 2mcg/mL [8, 77] Timing of administration Concurrent nephrotoxins (i.e. vancomycin) [77] Contrast administration	12.2% for gentamicin in neonates [78] 11.5-60% for aminoglycosides in adults [8, 74, 77, 79]	*Prevention:* Once daily dosing [75] Consider using tobramycin instead of gentamicin since it has lower rates of nephrotoxicity [80, 81] Avoid midnight to 7 am administration [76]	No difference in need for renal support in no gentamicin vs. gentamicin treated infection endocarditis, 8 vs. 6% respectively [82]	4.6% mortality in a cohort of 201 critically ill patients [8] 51% recovery within 21 days of AMG associated AKI [77] Cohort of critically ill patients, mortality in AKI vs. non-AKI group was 44.5 vs. 29.1%, respectively. [74]
Acyclovir Nephrolithiasis/AKI	Older children [83] Obesity [55, 84, 85]	Volume depletion CKD	Rapid intravenous administration Dose dependent Longer duration of therapy [83] Length of hospital stay [83] Concomitant nephrotoxins [86]	12-48% crystal nephropathy with rapid intravenous bolus administration 0.27% AKI from oral acyclovir [87] 3.1-10.3% children developed AKI from intravenous acyclovir	*Prevention:* Hydration Slow intravenous administration Dose adjustment for CKD *Treatment:* Discontinuation Rehydration Hemodialysis		
Calcineurin Inhibitors [88] AKI/glomerular	Genetic variations in CYP3A4, MDR1, ACE, TGF-β, and CCR5 [89–93]			42% in non-renal allografts [88]	Reduce dose Calcineurin minimization Calcineurin replacement with mTor inhibitors		
Cisplatin [52, 94] AKI/tubular	Age African Americans	CKD	Concurrent nephrotoxins	58% in pediatrics [52] 43.5% in adults [94]	Minimize concurrent nephrotoxin exposure		49% with reduction in GFR, 71% with glucosuria, 67% with proteinuria over long term [95]
Colistin [96] AKI	Age Obesity			48% in overweight or obese patients [96]	Minimize concurrent nephrotoxin exposure Consider alternative agents		80% developed failure by RIFLE category [96] No statistically significant difference in hospital or 30 day mortality [96]
Ifosfamide [97, 98] AKI/tubular	Age	CKD Nephrectomy Tumor infiltration in kidney	Cumulative dose Method of administration Concurrent nephrotoxins (cisplatin, carboplatin)	50% in pediatric cancer patients [97]	Minimize concurrent nephrotoxin exposure	No dialysis requirement [98]	No resolution of injury [98]
Lithium Tubular/Glomerular		CKD	Duration of therapy	11.6-15% develop AKI [99, 100] 26.1% develop concentrating defect [99]	Discontinuation of drug	78% of patients with Scr ≥2.5 mg/dL at baseline required dialysis [101]	42.1% develop ESRD [101]
Protease Inhibitors Atazanavir Indinavir Nephrolithiasis/ AKI				Asymptomatic crystalluria in 20-67% [102, 103] Nephrolithiasis in 3% [103]	Prevention: Patients should drink a minimum of 1.5 L/day of water to prevent stone formation Periodic monitoring of renal function and screening for pyuria during the first 6 months of therapy and biannually Treatment Hold if patient develops nephrolithiasis until rehydrated [104] Discontinue the drug if patient experiences pyuria, AKI, hypertension or rhabdomyolysis [104]	No dialysis requirements	21% increased risk of CKD [105] 12% increased risk of CKD [105]
Proton Pump Inhibitors AKI	Age > 60 years [12]		Current users higher risk compared to past users Concurrent nephrotoxins (antibiotics or diuretics) [10]	8-32 per 100,000 person-years [11, 12, 106]	Discontinue drug Consider course of steroids [107]	No dialysis requirement reported	Spontaneous recovery after drug withdrawal [108]
Sulfamethoxazole/trimethoprim	None	DM HTN CKD [109]	Concurrent nephrotoxins Contrast dye	11-22% experience AKI [109, 110]	Discontinue drug	1% required dialysis	Complete recovery within 30 days
Tenofovir Tubular				12-22% with proximal tubular injury [5, 6] 0.5% experience a renal event [111] 0.3% experience renal failure [111] 0.3-2% fanconi syndrome [112]	Prevention: Biannual screening for proteinuria and glycosuria with urinalysis, Scr, serum phosphate in patients with eGFR of < 90 ml/min/1.73 m^2^ [104]	<2% require dialysis [113]	16% increased risk of CKD [105] May have partial or complete recovery within months to a year
Vancomycin [29, 37, 114–124] AKI	Age Obesity	Sepsis Hypotension CKD Active cancers	Trough concentrations > 15 ng/mL Doses greater than 4 g/day Duration of therapy Concurrent nephrotoxins (ACEI, acyclovir, aminoglycosides, amphotericin, colistin, piperacillin/tazobactam, vasopressor use)	5-43% [29, 36, 114–116, 118, 119, 125, 126]	Employ therapeutic drug monitoring and pharmacist consultation [41] Maintain trough concentrations to < 15 ng/mL [38] Maintain doses < 4 g/day Consider switching to alternative antibiotics such as telavancin or linezolid [39, 40] Avoid combination with piperacillin/tazobactam [119] Minimize concurrent nephrotoxin exposure	Dialysis 0–7.1% [119, 126]	Resolution 21–72.5% [114, 119, 126] Mortality 45% [126]
VEGF Inhibitors Glomerular			Dose related [127]	21-63% incidence of hypertension [127] Case reports of nephropathy.	Reduce dose ACE inhibitors and nitrates to treat proteinuria and hypertension Discontinue drug		33% resolution of injury after discontinuation of therapy [7]

Risk factors contributing to the development of DIKD include patient specific factors, disease specific factors and process of care factors (Table 1). Common risk factors include age, causal drug single and/or cumulative dose, underlying CKD and concurrent nephrotoxin exposures. In the case of hospitalized patients, our experience is that a retrospective evaluation of DIKD almost always reveals the prescription of additional nephrotoxins concurrent to the causal drug (i.e. ketorolac prescribed to a patient receiving gentamicin and vancomycin). Minimizing these exposures may mitigate the development of DIKD.

Assessing kidney function is critical to the dosing of drugs and mitigation of DIKD. An important patient specific risk factor is low serum Scr values due to reduced muscle mass, which may be age related or disease related (muscular dystrophy, spina bifida, etc.). This poses a challenge to assessment of kidney function using estimating equations. Pharmacists often “round” Scr values to an arbitrary threshold value in older patients or those with low Scr values to account for low muscle mass. This practice is inaccurate and may lead to drug dosing errors in certain populations [13–16]. Currently, KDIGO guidelines on drug dosing advocate using either Cockcroft Gault or MDRD equation for drug dosing [17]. Since the drug information from manufacturers submitted to the U.S. Food and Drug Administration still utilizes the Cockcroft Gault equation for estimates of kidney function and no prospective studies have been conducted on clinical outcomes of the various equations, we feel that either equation could be used in the absence of kidney disease.

Published reports of DIKD have not consistently evaluated cases for the presence of common AKI risk factors. Subsequently, risk factors specific to a causal agent have emerged but have not been validated in larger studies and across multiple drugs. As an example, drug interactions have emerged as an important risk factor for the development of AKI. Interactions leading to increased concentrations of anti-hypertensive medications, subsequent hypotension and AKI have been reported [18]. In a study by Gandhi and colleagues, the risk for hospitalization with AKI was compared in patients receiving a prescription for amlodipine and one of two macrolide antimicrobials, clarithromycin or azithromycin. Clarithromycin is known to inhibit cytochrome P450 3A4 isoenzyme, which is involved in the metabolism of amlodipine, whereas azithromycin does not interact to the same extent. The authors found co-prescription with clarithromycin was associated with an odds ratio [OR], 1.98 [95% CI, 1.68–2.34] compared to co-prescription with azithromycin [18].

Identification of general DIKD risk factors is central to the development of clinical risk scores for the prediction and minimization of risk. For example, the identification of risk factors for contrast-induced nephropathy has led to the development of risk scores and evaluation of preventative treatments [19–23]. This has great applicability to the clinical setting, where an electronic medical record (EMR) can calculate the risk score and cardiologists or radiologists can prescribe preventative measures. Additionally these risk scores may predict long-term outcomes [24, 25].

## Recognition

Currently, there is no standard definition of DIKD and incidence of nephrotoxicity varies depending on the definition employed and the causal drug. The most common drugs that cause DIKD include antibiotics, anti-rejection medications, antiviral agents, non-steroidal anti-inflammatory agents, anti-ulcer agents and chemotherapy.

Most studies have defined nephrotoxicity as 0.5 mg/dL or 50% rise in Scr over 24–72 h time frame and a minimum 24–48 h of drug exposure. However, these definitions pose challenges since a 50% increase in Scr may not have high specificity for DIKD since the underlying disease being treated as well as other AKI risk factors could be significant to the attribution of risk. In the setting of fluctuating renal function or those patients receiving renal replacement therapies, it is difficult to recognize DIKD. For example, if a critically ill patient develops AKI from sepsis, it may be difficult to recognize whether an antibiotic is causing additional injury to the susceptible kidney. Recognition is also complicated by the fact that the mechanism of kidney injury and time period for onset of injury varies by drug and some drugs cause injury by more than one mechanism. For instance, NSAIDS can result in AKI due to hemodynamic changes or acute interstitial nephritis (AIN), or nephrotic range proteinuria from glomerular injury.

In order to improve the recognition of DIKD in the literature, we convened an expert panel to develop consensus-based definitions [26]. We propose that DIKD presents in one of four phenotypes: AKI, glomerular disorder, tubular disorder, or nephrolithiasis/crystalluria [26]. The clinical presentation of each phenotype is based on primary and secondary criteria. We suggest that at least one primary criterion must be met for all drugs suspected of causing DIKD [26]. For each phenotype definition, the following critical elements from the Bradford-Hill causal criteria must be met:

1. The drug exposure must be at least 24 h preceding the event.
2. There should be biological plausibility for the causal drug, based on known mechanism of drug effect; metabolism and immunogenicity.
3. Complete data (including but not limited to co-morbidities, additional nephrotoxic exposures, exposure to contrast agents, surgical procedures, blood pressure, urine output) surrounding the period of drug exposure is required to account for concomitant risks and exposures to other nephrotoxic agents.
4. The strength of the relationship between the attributable drug and phenotype should be based on drug exposure duration, extent of primary and secondary criteria met and the time course of the injury.

In defining the time course for DIKD, it is important to consider consensus definitions for AKI, acute kidney disease and CKD. Acute kidney injury develops in 7 days or less, injury beyond 7 days but less than 90 days reflects acute kidney disease and beyond 90 days CKD [27]. Using the KDIGO definitions, the development of DIKD can similarly be divided into acute (1–7 days), sub-acute (8–90 days) and chronic (>90 days) post drug exposure [26]. This approach permits classification and tracking of injuries for duration and outcomes. Based on this conceptual model, for each phenotype, thresholds could be established to detect DIKD, define its severity and ascertain recovery.

The reference Scr used for defining DIKD should as close as possible to the event to meet the definition of AKI but may not always be available as in the case of ambulatory care exposures. In this scenario, we recommend using the lowest Scr within 90 days of the event as the reference Scr. It is recognized that CKD is an important risk factor for the development of DIKD. Underlying kidney disease impacts the recognition of DIKD. We recommend using a Scr value greater than 90 days from the DIKD event to define the presence of CKD.

These standard definitions will become increasingly important when designing tools within the EMR to screen for DIKD. Such screening tools have been successful at identifying AKI and guiding the physician on the need for nephrology consultation [28]. Pharmacovigilence programs can identify patients who have been exposed to nephrotoxic medications and develop AKI with high serum drug concentrations [29]. Additionally, these electronic screening tools can be customized. At risk patients, such as those receiving multiple nephrotoxins or prolonged nephrotoxin exposures, can be targeted. Identification of such patients can prompt interventions such as intensified Scr monitoring and improve the recovery of DIKD [30]. However, electronic screening and identification cannot establish causality. These tools are limited due to the complex interplay of risk factor assessment, concurrent multi-drug exposures, lack of TDM, comorbid conditions and lack of kidney damage biomarkers. It is important that DIKD cases are adjudicated for causality and an attribution of risk is estimated for each contributing drug or risk factor. In the case of vancomycin, a pharmaco-vigilence program identified 32% of patients exposed to vancomycin with high trough concentrations and AKI [29]. However, when the cases were adjudicated, only 8.4% of AKI cases were attributed to vancomycin toxicity [29]. Attribution of risk from each potential risk factor or from each causal drug in the case of multi-drug injury is difficult since these assessments are based on the individual patient presentation and might reflect a substantial degree of subjectivity depending on the adjudicator’s knowledge of DIKD and AKI epidemiology. We recommend when evaluating cases of DIKD, the consulting nephrologist document their causality assessment in the medical record including a percent attribution assigned to each causal drug with an overall likelihood to cause the DIKD, as well as a percent attribution for each of the identified concurrent AKI risk factors. Adverse event causality scoring tools exist for general adverse events as well as drug induced liver and skin injury (Naranjo, Rucam, Liverpool), however, these tools have not been evaluated for the causality scoring of DIKD. Previous genomic studies of drug induced liver and skin injury have employed adjudication of cases by unbiased hepatologists or immunologists/dermatologists, respectively [31, 32]. Often, published case reports lack the evaluation of causality using these scoring systems or adjudication. As the body of knowledge surrounding DIKD increases, we recommend employing these scoring tools in addition to adjudication of cases by a secondary nephrologist when publishing case reports or series.

## Response

Treatment of nephrotoxicity is dependent on the phenotype, severity of the injury and the underlying condition for which the medication was prescribed. The decision to stop or reduce the dose of the offending drug requires a careful consideration of the risk versus benefit. In Type A reactions, dose reduction may be sufficient to mitigate the injury (e.g. vancomycin or gentamicin). However, stage 2 AKI often warrants drug discontinuation. In the setting of vancomycin DIKD, a critical appraisal of other therapeutic options and dose minimization is warranted. National guidelines on the use of vancomycin have recommended higher target trough concentrations to obtain a high area under the curve (AUC) to minimum inhibitory concentration (MIC) ratio [33, 34]. However, the level of evidence for this recommendation was grade IIIb (limited evidence) [34]. With the widespread adoption of this recommendation [35], the rate of nephrotoxicity has increased. Meta-analysis conducted found the incidence of nephrotoxicity to be between 5-43% and target trough concentrations > 15 ng/mL to have a 2.67 odds ratio for the development of nephrotoxicity [36]. A more recent study of 1430 patients receiving vancomycin provides support for the association between concentrations and duration of therapy with risk of nephrotoxicity [37]. Post hoc analysis of prospective studies have examined the need for higher targets and demonstrated equivocal or lower cure rates with trough concentrations above 15 ng/mL for the treatment of staphylococcus aureus nosocomial acquired pneumonia [38, 39]. Additionally, these studies have demonstrated that alternative treatments such as linezolid or telavancin could be considered [39, 40]. Based on these studies, we believe that DIKD from higher vancomycin trough concentrations is a real concern. However, prospective studies designed to evaluate the benefits and risks of high therapeutic concentrations need to be done. Type B DIKD, which is idiosyncratic, will require discontinuation of the offending drug and careful observation. Severe injuries or type B reactions often require longer periods of time to improve and may not completely resolve.

When DIKD has been identified, the patient should be monitored carefully including daily assessment of Scr and urine output as changes in kidney function may lead to further injury or lack of clinical cure for infections. Concurrent risk factors for kidney injury should be addressed such as but not limited to hypotension, hyperglycemia, anemia, minimization of nephrotoxins or drug interactions, which may contribute to the injury. Dose adjustments for kidney function should be made for other medications the patient is receiving. In some cases, timed urine collections for CLcr determination may be warranted to assist in the determination of renal function for the purpose of dosage adjustment. Where available, TDM should be employed and continued even after drug discontinuation in cases where supra-therapeutic concentrations are documented during the injury. Pharmacist consultation improves the achievement of target concentrations and improves clinical cure rates [41]. Additionally, documentation of the event is imperative to prevent future injuries from subsequent exposures. Patients should be informed of the event to empower them to inform other healthcare providers of their susceptibility to the drug.

Often, the sub-phenotype is difficult to distinguish from laboratory parameters (i.e. ATN vs. AIN) and kidney biopsy information can guide treatment decisions. Several studies have demonstrated the importance of kidney biopsies for classifying the type of injury and establishing the causal drug in the setting of nephrotoxicity. Zaidan and colleagues published a series of 222 kidney biopsies from HIV infected patients, 59 cases demonstrated tubulopathy or interstitial nephritis with 52.5% attributable to a drug [42]. Tenofovir was identified as the most common culprit of tubular damage in this series whereas infections and dysimmune syndromes accounted for the majority of interstitial nephritis cases [42]. Xie and colleagues published a case series of kidney injury from clindamycin, a previously unrecognized adverse event [43]. Biopsy results documented the majority of cases with AIN (75%) and remainder with ATN (25%) [43]. Chu and colleagues demonstrated that only 79.2% of patients with biopsy proven acute tubular necrosis met the clinical criteria for AKI [44]. Most patients had a slower increase in Scr than current KDIGO definitions [44]. Kidney biopsy information in addition to careful consideration of the temporal and causal relationship to drugs can provide a more accurate diagnosis of DIKD. Additionally, DIKD is often caused by multiple drugs and determining causality can be difficult. Even with kidney biopsy data, it may be difficult to determine exact causality for multi-drug injury. Sequential discontinuation of suspected causal drugs and subsequent re-challenge may assist in causality assessment.

## Renal Support

The need for renal support to treat DIKD is low (Table 1). The use of renal replacement therapy for DIKD is two-fold, firstly, dialysis can be utilized to remove the offending drug and minimize ongoing damage; additionally, dialysis can be utilized to support renal function to allow recovery. The decision to start renal replacement therapy is a complex one and generally reserved for severe injuries or cases in which the drug toxicity may be mitigated through removal by dialysis (example: vancomycin [45], aminoglycosides [46]).

Drug removal by dialysis is dependent on the characteristic of the drug including molecular weight, protein binding, volume of distribution and operational characteristics of the dialysis treatment include type of membrane, blood and dialysate flow rates and duration of therapy [47].

When starting renal support therapy, it is critical to consider the potential for errors in drug dosing and exposures. Renal clearance is determined by the dialysis prescription for drug dosing. However, in the critically ill population, gaps in dialysis delivery are frequent (e.g. filter clotting, time off treatment for procedures) and current prediction equations do not account for these situations. When possible, TDM should be employed. The decision to stop renal support is challenging and generally based on changes in pre-dialysis Scr values, urine output, fluid status and acidosis. These transitions from AKI with no renal support to dialysis therapies to resolution of injury are time periods with increased susceptibility to drug dosing errors. During recovery, total renal clearance should be estimated by quantifying intrinsic kidney function in addition to the dialysis therapy for drug dosing. Urinary creatinine clearance determination, despite its limitations [48, 49], may assist in the estimation of intrinsic kidney function. During resolution, drug clearance estimated from TDM may be used in the assessment of kidney function. For example, the clearance of aminoglycosides and vancomycin correlate well with creatinine clearance [50, 51]. Estimation of clearance of these drugs by TDM may assist the clinician in dosing other drugs that are not monitored (e.g. cephalosporins, quinolones).

## Rehabilitation

Most cases of nephrotoxicity are acute, non-oliguric and resolve with discontinuation of the causal drug (e.g. aminoglycosides) (Table 1). However, for some drugs, mixed patterns of injury may complicate the assessment of recovery. In the case of cisplatin, glomerular filtration decline tends to be reversible whereas tubular dysfunction may persist [52]. Clinical issues to consider include follow-up in specialized AKI ambulatory clinics and repeat assessment of kidney function to ascertain reversibility and delayed recovery, reporting of the adverse event to the U.S. Food and Drug Administration (https://www.accessdata.fda.gov/scripts/medwatch) [53], documentation of the adverse effect in the allergy section of the EMR, limiting re-exposures to the causal drug and appropriately using information from past exposures (i.e. TDM) to limit future adverse events. For example, TDM software can be used to determine appropriate initial dosing for antibiotics based on population based pharmacokinetic parameters. However, if a patient has experienced AKI from an antibiotic in the past and the information is relatively recent, the past pharmacokinetic parameters estimates for that particular patient should be used to guide future empiric dosing recommendations. Therapeutic drug monitoring may prevent nephrotoxicity and result in cost avoidance [54, 55].

## Research

Research in the epidemiology of DIKD has been limited. The lack of consensus definitions has led to variability in the incidence of DIKD. Acute kidney injury is multifactorial and risk factors for AKI have been identified for different populations. Risk factors for DIKD vary by drug; however, it is very likely that we can determine common risk factors that identify vulnerable populations, such as older age or history of CKD. Establishing causality is challenging in DIKD; it requires attribution of risk to not only the suspect drug but also the relative contribution from each concurrent risk factor. Newly identified DIKD is often published as case reports or case series. In such reports, the attribution of risk from concurrent risk factors is lacking. Clinical trials and FDA surveillance programs provide some information, but post-marketing surveillance is voluntary and requires clinicians to identify the association of an AKI event with a drug. As accepted definitions of AKI are implemented in research and AKI awareness increases, the identification and outcomes of DIKD will be better characterized. The use of EMRs and decision support tools will facilitate electronic surveillance of DIKD, identifying the event and risk factors and directing clinicians on treatment options. However, more research is required in the area of causality assessment, risk score calculation and adjudication of cases. Electronic surveillance alone provides an initial step in case identification, however, without causality assessment, these tools lack validity and will lose their effectiveness in clinical practice.

Often, histologic confirmation of drug toxicity is lacking. The decision to biopsy a patient is a risk/benefit assessment and central is the question of whether a biopsy will change patient management? The majority of clinicians opt to discontinue a suspect drug rather than biopsy. However, given the lack of validated tools for causality assessment, we feel that biopsy information from cases series of drug toxicity will contribute significantly to this area of research. As our body of knowledge increases; risk score calculators, causality assessment tools coupled with information on outcomes of DIKD can lead to the development of predictive analytics which may identify individuals at risk of nephrotoxicity from a drug. Population based studies provide a large sample and aid in the identification of rare or previously unrecognized events such as antipsychotic associated AKI [56] or drug interactions that lead to drug toxicity and AKI [18].

Translational research models of drug-induced nephrotoxicity identifying pharmacokinetic parameters, drug transporters and kidney injury biomarkers have been developed but there are no prospective studies validating these models [57–61]. Molecular characterization of drug toxicity using proteomics and microarrays will further delineate mechanisms for kidney injury and pathways for repair [62–65]. Ongoing research in the area of biomarkers to earlier detect damage in drug toxicity will facilitate prevention of functional changes [66–70].

Importantly, research on the preventative strategies and response to nephrotoxicity is limited. Although, TDM and pharmacokinetic analysis improves achievement of target concentrations, vulnerable populations do not have the same pharmacokinetic parameters as general population based estimates. For example, there is limited information on pharmacokinetic parameters for specific nephrotoxic drugs in CKD or heart failure putting these populations at risk. Additionally, TDM is not uniform across the United States. There is a need for increased pharmacokinetic studies on vulnerable populations, as well as studies on target drug concentrations linked to patient outcomes. The emergence of increased vancomycin nephrotoxicity is a case example of widespread application of consensus-based recommendations on higher drug concentrations with a lack of strong evidence for patient outcomes across various types of infections.

Consensus guidelines on drug dosing in AKI provide practical recommendations and should be considered for dosing other drugs that patient is concurrently taking during a nephrotoxic insult [17]. However, research on drug dosing in AKI is limited. More information is needed on kidney function estimation in AKI, the impact of tubular function and metabolism of drugs during AKI and pharmacokinetic changes in AKI [71–73]. Enhancing this research will translate to the mitigation of additional risk and in turn enhanced recovery from a nephrotoxic event.

The field of AKI and DIKD research is rapidly evolving with the development of large international registries of patients with AKI and DIKD. This presents tremendous opportunities for trainees with an interest in kidney disease to collaborate with other disciplines on research that will enhance our body of knowledge in these important areas.

## Conclusions

In conclusion, we provide a 6R framework for DIKD that allows clinicians to apply what is known about DIKD and more importantly to recognize the unknown and limitations of our current clinical care.

## Abbreviations

AIN: Acute interstitial nephritis
AKI: Acute kidney injury
ATN: Acute tubular necrosis
AUC: Area under the curve
CKD: Chronic kidney disease
CLcr: Clearance creatinine
DIKD: Drug induced kidney injury
EMR: Electronic medical record
KDIGO: Kidney disease improvement global outcomes
MIC: Minimum inhibitory concentration
NSAIDs: Non-steroidal anti-inflammatory drugs
Scr: Serum creatinine
TDM: Therapeutic drug monitoring
